# Effect of *Yoganidra* on Blood Pressure, Hs-CRP, and Lipid Profile of Hypertensive Subjects: A Pilot Study

**DOI:** 10.1155/2021/2858235

**Published:** 2021-12-30

**Authors:** J. P. Devraj, B. Santosh Kumar, M. Raja Sriswan, B. Jagdish, B. S. Priya, S. B. Neelu, Vijayabhaskar Desai Rao, Manoj Kumar, J. J. Babu Geddam, R. Hemalatha

**Affiliations:** ^1^Division of Clinical Epidemiology, ICMR-National Institute of Nutrition, Hyderabad, Telangana-500 007, India; ^2^Clinical Division, ICMR-National Institute of Nutrition, Hyderabad, Telangana-500 007, India; ^3^Behavioral Science Unit, Extension & Training Division, (ICMR)-National Institute of Nutrition, Hyderabad, Telangana-500 007, India; ^4^ICMR- National Institute of Nutrition, Hyderabad, Telangana-500 007, India; ^5^Department of Biostatistics, (ICMR)-National Institute of Nutrition, Hyderabad, Telangana-500 007, India; ^6^Department of Microbiology, ICMR-National Institute of Research in Environmental Health, Bhopal, Madhya Pradesh-462030, India

## Abstract

**Background:**

*Yoganidra* is a systematic method of promoting a state of complete physical, mental, and emotional relaxation. It is a safe, inexpensive, and very effective method of management of hypertension when used along with standard pharmacological therapy. This study aims to assess the effect of *yoganidra* on blood pressure (both systolic blood pressure (SBP) and diastolic blood pressure (DBP)), Hs-CRP, and lipid profile of hypertensive subjects at the time of enrollment (subjects that are hypertensive at the time of enrollment).

**Methods:**

Both treated and untreated subjects (*n* = 74) with hypertension (blood pressure ≥140/90 mmHg) and age between 35 and 70 years were included in this study after obtaining ICMR-NIN-IEC approval and written informed consent from all subjects. Subjects with critical illness and/or psychological disturbances were excluded from this study. The subjects in the experimental group (*n* = 31) practiced *yoganidra* for 45 minutes daily for 12 weeks under strict supervision. There was no intervention in the control group (*n* = 43). Weekly blood pressure was recorded in the experimental group, whereas it was performed at baseline and at endpoint for control groups. Hs-CRP and lipid profile were estimated at baseline and endpoint for both the groups.

**Results:**

A significant reduction in mean SBP from 142.9 mm Hg (SD ± 16.46) to 118.68 mm Hg (SD ± 9.21; *p* value 0.0001) and DBP from 89.84 mm Hg (SD ± 10.42) to 77.03 mm Hg (SD ± 6.47: *p* value 0.0001) was observed among the experimental group after 12 weeks of *yoganidra* practice when compared with the control group. A significant reduction in mean Hs-CRP (2.21 ± 1.49 to 1.06 ± 0.82 mg/L, *p* < 0.001^*∗∗∗*^) was observed among the experimental group. There were no significant differences between triglycerides and total cholesterol levels, whereas LDL-C and HDL-C showed a trend of improvement in the experimental group after intervention.

**Conclusions:**

In this pilot study, we observed a significant reduction in blood pressure and Hs-CRP in the *yoganidra* group compared with the control group. There were no significant side effects observed in the intervention group during the study period.

## 1. Introduction

Hypertension (HTN) is a significant public health problem, which affects the cardiovascular health of most of the people in India. According to the World Health Organization [1], 140/90 mm Hg or higher is considered hypertension. By 2025, there will be 1.56 billion adults who may become hypertensive, as globally estimated. Nearly 8 million and 1.5 million people will die every year worldwide and in the South-East Asia (SEA) region [1], respectively. The prevalence of hypertension in India is seen to be increasing day by day. One among the three adults is reported to be hypertensive in a recently shown national-level survey. It was conducted with a fixed one-day blood pressure measurement of 180,335 participants through camps across more than 20 states of India. A study reported that the prevalence of hypertension was 30.7% [2]. A recent “Global Burden of Disease” study reported that hypertension led to India's 1.63 million deaths in 2016 alone. It was the second leading risk factor in terms of attributable disability-adjusted life years (DALYs) in men (122.2 million DALYs) after smoking and the leading risk factor in women (89.9 million DALYs) [3]. Nevertheless, about 25.6% of subjects on medication had their B.P. under normal levels. More than 50% of all stroke deaths and 24% of all coronary heart disease deaths in India are due to raised blood pressure [4]. Long-term high blood pressure leads to coronary artery disease, stroke, heart failure, atrial fibrillation, peripheral vascular disease, vision loss, chronic kidney disease, and dementia [5, 6]. Thus, the burden of hypertension can be genuinely considered as a pandemic. It has been found that dyslipidemia, inflammation, stress, high-risk behaviors, and sedentary and unhealthy lifestyles are significant causes of hypertension. Dyslipidemia is recognized as a prominent risk factor for CVD [7]. Altered lipid parameters, low HDL levels, raised LDL-C, and triglycerides are associated with a rise in cardiovascular diseases. Hs-CRP is an acute-phase reactant synthesized by the liver and elevated in response to acute infections, inflammatory conditions, and trauma [8]. Several studies have shown that serum Hs-CRP can be an excellent diagnostic and a prognostic marker in diagnosing prehypertensive subjects, and elevated levels have shown a greater risk of stroke and cardiovascular disorders [9–11]. Antihypertensive medications are essential in the management of hypertension. Major antihypertensive agents used are ACE inhibitors, diuretics, *β*-blockers, angiotensin II receptor blockers, calcium channel blockers, and vasodilators. Due to the side effects of these medications, nonadherence occurs in about 50% of newly treated hypertensive patients within the first year of treatment [12–15]. Long-term use of antihypertensive drugs has a significant risk of developing type 2 diabetes, osteoporotic fractures, atrial fibrillation, and breast cancer in women [16–18]. Over the years, patients with hypertension may experience mental health disorders such as anxiety, depression, and stress, which again lead to increased blood pressure [19]. Long-term use of medications leads to decreased quality of life [20]. All these constitute a significant burden on the economy of the country. Several studies have been conducted on diet and physical activity to reduce blood pressure in hypertensive subjects [21, 22]. Although these have been mainly beneficial, they tend to be effective as long as a given regimen is followed. Often people find it challenging to incorporate specific lifestyle changes. It is observed that *yoganidra*, when used along with standard therapy, is a safe, inexpensive, and very effective method of management of hypertension [23–26]. *Yoganidra* has been used earlier as a therapeutic option with no documented side effects. Previous studies have separately reported the effect of *yoganidra* on hypertension, Hs-CRP, and lipid profile [23–26]. In this study, we have assessed all the three parameters (reduction in blood pressure, Hs-CRP, and lipid profile) in one study among hypertensive subjects on pilot mode.

## 2. Methodology

### 2.1. Sample Size and Study Subjects

Since this study was planned to be a pilot one, we have not calculated the sample size for this study. The nonprobability convenient sampling technique was adopted to select the subjects for this study. Subject recruitment was performed based on the inclusion and exclusion criteria listed below.

### 2.2. Inclusion and Exclusion Criteria

Subjects (on medication/without medication) aged between 35 and 70 years old having blood pressure ≥140/90 mmHg were screened. Subjects who were pregnant and lactating, those who were with critical illness and hearing impairment, or those taking any kind of sedatives or psychological treatments were excluded from this study. Seventy-four eligible subjects with HTN were recruited after obtaining NIN-IEC approval (IEC no. 04/II/2016) and written informed consent. Data on anthropometry such as height, weight, sociodemographic details such as education, professional clinical history regarding the onset and duration of hypertension, family history, and duration of drug therapy, type, and dosage of drugs was obtained. The experimental (or test) group included subjects who could comprehend the relaxation techniques, showed interest in *yoganidra*, and could commit to attending and regularly practicing *yoganidra* as per our recommendation. Others were included in the control group and were asked to continue with a regular diet, physical activity, and medications prescribed by physicians. Blood samples (5 ml) were collected for Hs-CRP and blood lipid profile for all recruited subjects at baseline and endpoint (after 12 weeks). In the intervention group, thirty-one hypertensive subjects on medication (*n* = 19) and no medication (*n* = 12) were included to practice yoganidra daily (45 min/d) for 12 weeks, at institute premises under the guidance of a trained yoga instructor. During the study duration, blood pressure was monitored weekly by the physician before the *yoganidra* session—using both manual and digital B.P. apparatus. The subjects were asked to relax for 10 min before the start of *yoganidra* to decrease or minimize the white-collar effect.

### 2.3. Blood Pressure and Data Collection

Our main aim was to assess the impact of yoganidra on blood pressure in the intervention group and evaluate its effect on a weekly basis. We included the control group to compare their blood pressure at two time points with the intervention group. Blood pressure was measured in the sitting position before starting *yoganidra* every week for 12 weeks for all subjects in the experimental group using a digital blood pressure monitor (oscillatory-OMRON) and mercury sphygmomanometer (auscultatory). Each subject was asked to relax in a sitting posture for 5 min. Three readings of digital blood pressure monitor and one reading of mercury sphygmomanometer were taken to ensure accuracy. The first reading of blood pressure was measured using a digital blood pressure monitor. Two more readings were recorded by maintaining an interval of 5 minutes. The fourth reading was taken using a mercury sphygmomanometer. All readings were duly recorded.

### 2.4. MAP

Mean arterial pressure (MAP) is the average blood pressure in an individual during a single cardiac cycle. It is calculated by using the following formula MAP = SBP + 2DBP/3. MAP value of 70–100 is considered to be normal. Any variations, which are high or low, are a sign of the underlying medical disorder.

### 2.5. Control Group

Forty-two hypertensive subjects with or without medication were included in a control group without any lifestyle modification. One subject was excluded due to noncompliance. Both male and female subjects were included with disease duration ranging from 1 to 15 years.

### 2.6. Intervention Group

The yoganidra intervention was conducted at the institute premises in a semidark room from 5:30 pm to 6:30 pm for 5 days in a week for 12 weeks. The subjects were strictly monitored, and an attendance register was maintained to check the regularity of the subjects. Then, subjects were asked to do warm-up exercises like body rotation and joint rotation after a 2-3 min brisk walk to get them ready for *yoganidra* intervention. These warm-up steps help subjects to be active, attentive, and energetic during the intervention period. Otherwise, there is a possibility of subjects going into deep sleep. Then, subjects were asked to lay in *shavasana* (relaxed lying down on the back) position for *yoganidra* protocol (35 min) in the semidark room, under the supervision of the yoga instructor and an investigator. The room was provided with an audio CD player and speakers. Since darkness and light have an effect on the brain, semidarkness is necessary to maintain a state of relaxed awareness to maintain a balance between introversion and extroversion.

### 2.7. The Technique of *Yoganidra*

*Yoganidra* means sleeping consciously, which systematically induces complete physical, mental, and emotional relaxation [26]. *Yoganidra* has a preventive, promotive, and curative value. It is a noninvasive, easy-to-practice, cost-effective intervention to prevent stress [24]. *Yoganidra* is a meditation and relaxation technique that focuses more on the mind than on the physical body. Its mode of principal action on the mind may bring down sympathetic activity and reduce blood pressure [24].

### 2.8. Stages of Yoganidra

The practice of yoganidra was performed in the following stages: in the preparatory stage, body and mind were wholly relaxed by inducing the awareness of stillness and comfort of the body by correcting posture and position, speed of breath, and listening to the external sounds. The subjects were educated to be aware of the surroundings and asked them to be in the state of witnessing the activity. In the second stage, subjects were instructed to take *sankalpa* or a resolution according to their wish after the body and mind were completely relaxed. They were told to keep it short, clear, and positive and were asked to mentally repeat the selected *sankalpa* three times, with full determination, conviction, and confidence. During the third stage, consciousness or awareness was systematically switched throughout the different parts of the body. Subjects were instructed to remain aware, listen to the instructions, and very rapidly move the mind according to the instructions without making any physical movements. A definite sequence was followed to shift the awareness. In the next stage, subjects were asked to become conscious of the natural breath without changing the breath flow. Subjects were aware of each inspiration and expiration by mentally counting them. During the 5th stage, the physical or emotional sensations were recalled, intensified, and fully experienced. Pairs of contradictory feelings or sensations were practiced by asking subjects to imagine the heat and cold, heaviness and lightness, pain and pleasure, love and hate, and so on. In the visualization stage, the subjects were asked to visualize some objects, stories, or situations in the *chidakasha* (“space of consciousness” or “inner space.”) At this time once again, subjects were asked to mentally repeat *sankalpa* three times, which was taken earlier in stage two, with full dedication, faith, and optimism. In the final stage, slowly, the awareness was externalized by asking the subjects to become aware of the external sounds, objects, and persons. They were then asked to slowly move the body parts and to stretch the body. We observed that initially, for 3–5 days, most of the subjects fell asleep for 5–10 min. It was difficult for them to remain awakened during *yoganidra*. After five days, they got used to the practice of *yoganidra*.

### 2.9. Quality Control

As the first step, midupper arm circumference was measured to use an accurate cuff bladder size to obtain accurate blood pressure. Quality control for measuring blood pressure was ensured, which includes two parts, the checking of equipment and performance monitoring of the blood pressure recorders. Before checking the first blood pressure measurement, we checked to ensure that the sphygmomanometer's mercury column was at zero and the mercury column smoothly falls when the cuff was deflated. We also checked whether the column properly latches into the vertical position. The results were recorded in a book. The following precautions were taken for the correct measurement of blood pressure: an average of three readings was taken at intervals of 5 min. The subjects were allowed to sit for at least 10 min in a quiet room before taking blood pressure measurement.

Blood pressure measurement was performed in a standard sitting position. The subject's arm was fully bared and supported at the level of the heart. The blood pressure recordings were taken as mentioned in the intervention group. These readings were taken as pretrial blood pressure measurements. The controls were selected from our institute's clinic, and 62 subjects were screened, out of which, 43 subjects were selected based on inclusion and exclusion criteria. In this group, 30 men and 13 women were included with hypertension duration ranging from 1 to 20 years with a mean of 5.02 years. Thirty-four were on antihypertensive drugs, and the remaining subjects were newly diagnosed, who are on diet and lifestyle modification. All the recruited subjects were asked not to change their drug and dosage during the study period. None practiced *yoganidra* earlier, both among the intervention and the control group.

### 2.10. Biochemical Parameters

Five ml blood samples were collected (twice) from all recruited subjects in the morning after an overnight fast of 12 hours to assess the effect of *yoganidra* on Hs-CRP and blood lipid profile.

### 2.11. Hs-CRP

Hs-CRP was measured by immunoturbidimetry. It was classified according to the recommendation of the American Heart Association and Center for Disease Control, defining Hs-CRP <1 mg/L as low risk, between 1 and 3 mg/L as moderate risk, and >3 mg/L as high risk [27].

### 2.12. Lipid Profile

Total cholesterol and triglyceride concentrations were determined by enzymatic methods. HDL-C was determined following the deletion of triglyceride-rich lipoprotein and low-density lipoprotein (LDL-C) using the HDL direct liquid select TM kit. LDL cholesterol was calculated using Friedewald's formula: LDL-C = TC-HDL minus (TG/5) [28].

### 2.13. Statistical Analysis

Descriptive statistics was performed employing the SPSS-20 version for variables with a normal distribution.

Descriptive values were expressed as means and standard deviations. In this study, *p* < 0.05 was considered to be the level of significance. The proportions of responders and nonresponders to *yoganidra* were compared among treated/on medication and untreated/no medication groups by percentage.

## 3. Results

**Figure 1:**
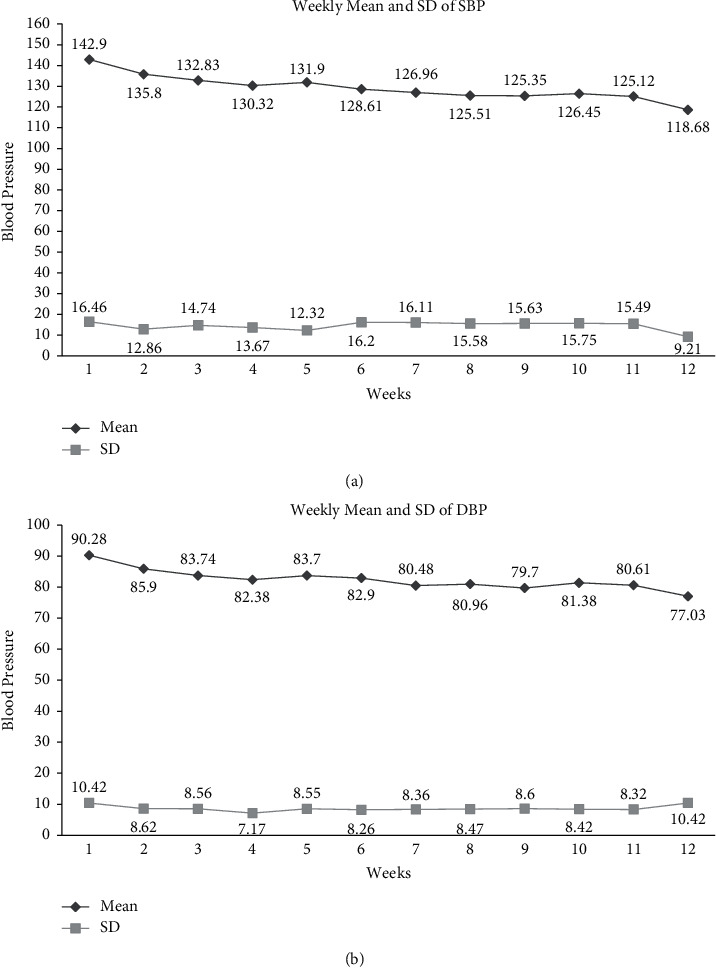
(a) Weekly mean and SD of SBP among the yoganidra-treated group and (b) weekly mean and SD of DBP among the yoganidra-treated group.

Both treated and untreated subjects (74) with hypertension were recruited and were grouped into the experimental (*n* = 31) and control group (*n* = 43). Anthropometry and sociodemographic details are as shown in Table 1. Male subjects were more among both the groups (77% in the experimental and 70% in the control group). The mean age of study subjects in the experimental group was 54.61 years (SD ± 9.95) and the control group was 49.64 years (SD ± 7.67). More than 95% of subjects were aged more than 45 years in the experiment, whereas it was 80% among the control group. About 90% of subjects were literate up to intermediate level and above among both the groups. Less than 10% had a temporary job, and 10% were unemployed. The remaining subjects were either government employees or were engaged in business among the experimental group, whereas 14% were unemployed and 21% had temporary jobs among the control group. Comparisons of BMI, SBP, DBP, PR, MAP, Hs-CRP, and lipid profile are shown in Table 2. Significant reduction in mean SBP from 142.9 mm Hg (SD ± 16.46) to 118.68 mm Hg (SD ± 9.21) (*p* value 0.0001) was observed among the experimental group after 12 weeks of yoganidra intervention when compared with the control group (from baseline: 134.29 mm Hg (SD ± 14.17) to endpoint: 130.71 mm Hg (SD ± 16.21: *p* value 0.114)). A significant reduction in DBP was observed among the experimental group from 89.84 mm Hg (SD ± 10.42) to 77.03 mm Hg (SD ± 6.47: *p* value 0.0001) after 12 weeks of *yoganidra* practice when compared with the controls (baseline: 86.84 mm Hg (SD ± 14.17) to endpoint 84.16 mm Hg (SD ± 9.82: *p* value 0.121)). The mean difference and median of SBP were 24.22 and 23.00, whereas the mean difference and median of DBP were 12.8 and 14.0 after 12 weeks of the intervention of yoganidra among the experimental group. Since SBP and DBP were not matched at baseline, after adjusting for them, a highly significant reduction in SBP (mean 16 mm/Hg) and DBP (mean 8.6 mm/Hg) was observed among the experimental group (*p* < 0.001^*∗∗*^). Weekly blood pressure was recorded for experimental group subjects, and the mean and SD of both SBP and DBP were as shown in Figures 1(a) and 1(b). A gradual reduction in both SBP and DBP was observed among the intervention group. Furthermore, when the effect of *yoganidra* was compared among treated/on medication and untreated/no hypertensive medication subjects (Table 3), there were 19 subjects on standard pharmacological treatment, whereas 12 were newly diagnosed untreated cases in the experimental group. The mean difference of SBP among treated and untreated groups was 23.47 and 27.03 mm Hg and DBP was 11.52 and 15.57 mm Hg, respectively. It was observed that the mean difference between SBP and DBP was more among the untreated group. Any intervention that reduces blood pressure by 10 mm Hg SBP and 5 mm Hg DBP has good clinical significance [29]. We grouped among responders (SBP >10 mm hg and DBP >5 mm hg) and nonresponders. When the proportions of responders and nonresponders were compared (Table 4), which showed that SBP responders were 94.73% in the treated group and 83.33% responders were in an untreated group, whereas for DBP nonresponders were 79% in treated and 100% in the untreated group. MAP was compared from baseline to endpoint among both groups. A significant reduction in MAP (107.76 ± 11.07 to 90.92 ± 6.48: mean difference 16.69 *p* values 0.0001) was observed among the experimental group, whereas reduction in MAP was not significant in the control group (102.58 ± 9.44 to 99.75 ± 11.23). When Hs-CRP was analyzed, a significant reduction in mean Hs-CRP (2.21 ± 1.49 to 1.06 ± 0.82 mg/L; *p* < 0.001) was observed among the experimental group after 12 weeks of a regular practice of *yoganidra,* whereas with a significantly increased mean Hs-CRP was observed among the control group (1.22 ± 1.05 to 2.57 ± 1.71 mg/L; *p* < 0.001). When the lipid profile was analyzed, it was found that there were no significant differences in triglycerides and total cholesterol levels among both groups, whereas HDL-C and LDL-C showed a trend of improvement without statistical significance in the experimental group after the intervention, which may be due to the small sample size.

## 4. Discussion

Hypertension is an important and common risk factor for cardiovascular diseases, stroke, diabetes, etc., which leads to considerable morbidity and mortality in developed and developing countries and is a pandemic problem. Hypertension and its ancillary complications globally are a leading cause of death in modern societies. The medications used in the management of hypertension not only decrease blood pressure but also cause some side effects [20] that lead to poor compliance. Prolonged usage of medications may cause the onset of chronic noncommunicable diseases, decreased quality of life, and work output, thus adding a major burden on the country's economy. Hypertension is a multifactorial disease, and its pathogenesis is not fully understood. In subjects with hypertension, arterial pressure is persistently high without any identifiable cause. It is mainly dependent on cardiac output and total peripheral resistance. The possible mechanisms are believed to be sympathetic nervous system overactivity and consequent increase in peripheral vascular resistance. Besides this, the sympathetic nervous system's direct pressure effect and catecholamine released from the adrenal medulla may also play a role. Hypertrophy of systemic arterioles could represent an adaptive response to chronically elevated blood pressure and perpetuate systemic hypertension. Inappropriately high sympathetic nervous outflow from the central nervous system is also believed to be an important component in the pathophysiology of acute and chronic essential hypertension, increasing cardiac output, and peripheral resistance [30].

*Yoganidra* centrally acts upon the brain to induce complete relaxation throughout the nervous system, and it improves the resistance levels of the physiological and physical systems of an individual. It controls the autonomic nervous system, influencing the brain's electrical rhythms, heart rate, and systolic and diastolic blood pressures. It maintains altered levels of circulating “stress hormones” including adrenaline and cortisol from the adrenal glands and decreased sympathetic nervous activity as reflected in increased galvanic skin resistance. In this pilot study, we used *yoganidra* as adjuvant therapy along with the standard medications. A significant reduction in SBP and DBP was observed after 12 weeks of supervised yoganidra practice in hypertensive subjects. Our observations are in line with earlier studies [23–25, 31–35]. Even though there was a gradual reduction in both SBP and DBP from baseline to endpoint. During the 4th week to the 5th week, there was a slight increase in both SBP and DBP, which was shown in Figures 1(a) and 1(b), which may be due to the fact that the subjects reduced their dose of antihypertensive drugs by 50%. A significant reduction in SBP and DBP and a high response rate among the untreated (Tables 3 and 4) compared with the treated group suggests that *yoganidra* is very effective among untreated or newly diagnosed subjects with HTN. To confirm our observations and recommend *yoganidra* to hypertensive subjects, multicentric randomized controlled trials need to be planned. A significant reduction in MAP was observed among the *yoganidra* group compared with the control group, which is reported for the first time. It was normalized in our study subjects after 12 weeks of *yoganidra* practice. Hs-CRP and erythrocyte sedimentation rate (ESR) are known as inflammatory markers with clinical significance among hypertensive subjects. In this study, a significant reduction in Hs-CRP levels among the intervention group was observed compared to the control group. There are no studies so far on the effect of *yoganidra* on Hs-CRP among hypertensive subjects. In contrast, the earlier study by Kumar and Pandya [35] suggested that *yoganidra* has the potential to reduce ESR levels among normal subjects, which proved that *yoganidra* may have a significant role in controlling infection and inflammation and improving immunity. In this study, we have observed a trend of improvement of HDL and LDL levels, whereas no significant changes were observed on triglycerides and total cholesterol levels after yoganidra intervention. Less number of subjects and a short duration may be the reason for the same. The uniqueness of this pilot study is that no study in the recent past had implemented a supervised *yoganidra* intervention with a comparison between an experimental and a control group, weekly blood pressure monitoring, and monitoring the participants for Hs-CRP and lipid profile. Many subjects showed definite symptomatic improvement after 12 weeks of *yoganidra* with no side effects. We conclude that this therapy opens a new avenue in the management of hypertension. We suggest that *yoganidra* may be used as a stand-alone therapy for untreated/asymptomatic for early or newly diagnosed hypertensive or prehypertensive subjects that may not require any medications to manage hypertension. There are separate reports, which show that the *yoganidra* impacts psychological well-being, quality of life, blood sugar levels, mental health (stress, concentration, and behavior), and sleep disorders [31–35]. Overall, *yoganidra* can be considered as an add-on option for the prevention and control of chronic noncommunicable diseases in all age-groups, which can be regularly practiced at home, at the office, and community centers, without much financial burden. *Yoganidra* can be incorporated with the “Fit India” movement along with other physical exercises. Most of our population working in government and private organizations are seen to be suffering from type 2 diabetes, hypertension, dyslipidemia, depression, and stress, which affect their work efficiency, leading to financial losses not only to the individual but also to the county as well. *Yoganidra* can be regularly practiced after office hours, which can prevent and manage hypertension, cardiovascular events, and improve mental health status without medications. Additionally, no side effects were observed in practicing *yoganidra*, and this could be effectively incorporated as an indigenous novel intervention for newly diagnosed hypertensive and psychosomatics under the National Health Scheme.

## 5. Conclusions

In this pilot study, we observed a significant reduction in SBP, DBP, and MAP among the *yoganidra* intervention group as compared with the control group. *Yoganidra*, one of the components of yoga, should become an integral part of human life. The good health of people always impacts the country's economy in a positive way.

## 6. Recommendations

Keeping in view the significant findings of this study and the potential beneficial impact of *yoganidra* on managing HTN and related health issues, it is highly recommended to carry out multicentric randomized controlled trials with a larger sample size categorized by pharmacological groups. Other sympathetic parameters may be evaluated for a better understanding of the pathophysiology involved in reducing hypertension. Thus, the data may help us bring policy reform to address the burden of hypertension in the country.

## 7. Limitations

The small sample size is a significant limitation of this study. However, this pilot study provides significant impetus to initiate major trials to evaluate the positive effect of *yoganidra* on hypertension and other NCD markers.

## Figures and Tables

**Table 1:** Comparision of demographic details among both groups.

	Experimental (*n* = 31)	Control (*n* = 43)
Male (*n*+ %)	24 (77.4)	30 (69.8)
Female (*n*+ %)	7 (22.6)	13 (30.2)
Age (mean ± SD)	54.61 ± 9.95	49.645 ± 7.67
35–44 (*n*+ %)	1 (3.2)	9 (20.93)
45–54 (*n*+ %)	7 (22.6)	21(48.83)
55–65 (*n*+ %)	10 (32.3)	11 (25.58)
>65 (*n*+ %)	13 (41.9)	2(4.65)
Illiterate (*n*+ %)	1 (3.2)	4 (9.3)
Up to inter (*n*+ %)	7 (22.6)	21 (48.8)
Up to graduate (*n*+ %)	10 (32.3)	9 (20.9)
PG and above (*n*+ %)	13 (41.9)	9 (20.9)
Govt job	12 (38.7)	18 (41.9)
Pvt/temporary job	3 (9.7)	9 (20.9)
Business	5 (16.1)	9 (20.9)
Not earning	3 (9.7)	6 (14.0)
Retired personnel	8 (25.8)	1 (2.3)
HTN-T	19 (61.29)	34 (79.06)
HTN-U	12 (38.7)	9 (20.93)

**Table 2:** Effect of yoganidra on blood pressure, Hs-CRP, and lipid profile among experimental and control group subjects.

Sl. no.	Parameters	Experimental group (*n* = 31)				Control group (*n* = 43)			
		Pre (mean ± SD)	Post (mean ± SD)	Mean diff	*p* value	Pre (mean ± SD)	Post (mean ± SD)	Mean diff	*p* value
1	BMI	28.68 ± 3.91	28.29 ± 3.62	0.389	0.009^*∗*^	27.61 ± 4.77	27.58 ± 4.95	0.03	0.872
2	SBP	142.9 ± 16.46	118.68 ± 9.21	24.22	0.0001^*∗∗*^	134.29 ± 14.17	130.71 ± 16.21	3.58	0.114
3	DBP	89.84 ± 10.42	77.03 ± 6.47	12.8	0.0001^*∗∗*^	86.84 ± 10.13	84.16 ± 9.82	2.68	0.121
4	PR	80.61 ± 11.49	78.94 ± 9.05	1.677	0.247	84.16 ± 9.82	81.19 ± 11.73	−0.52	0.85
5	MAP	107.61 ± 11.07	90.92 ± 6.48	16.69	0.0001^*∗∗*^	102.58 ± 9.44	99.75 ± 11.23	2.83	0.072
6	Hs-CRP	2.21 ± 1.49	1.06 ± 0.82	−1.15	0.0001^*∗∗*^	1.22 ± 1.05	2.57 ± 1.71	1.35	0.0001^*∗*^
7	Trig	155.84 ± 195.56	158.71 ± 140.73	−2.87	0.858	131.24 ± 74.28	130.84 ± 54.63	0.4	0.971
8	Tot. Chol	184.94 ± 32.19	186.00 ± 33.65	−1.0645	0.806	175.04 ± 37.46	181.0 ± 34.86	−5.96	0.147
9	HDL	51.0 ± 11.93	53.97 ± 14.04	−2.968	0.359	52.64 ± 9.57	51.73 ± 12.50	0.91	0.77
10	LDL	95.83 ± 29.64	89.63 ± 28.40	6.2	0.297	92.76 ± 28.96	88.0 ± 31.90	4.76	0.384
11	VLDL	31.17 ± 39.11	31.74 ± 28.15	−0.574	0.858	26.25 ± 14.±86	26.17 ± 10.93	0.08	0.971

SD, standard deviation. ^*∗*^Significant and ^*∗∗*^highly significant (*p* value < 0.005).

**Table 3:** Effect of yoganidra on blood pressure among untreated and treated cases.

Sl. no.	Group	Parameters	Time points	*N*+	Mean (±SD)	Mean diff	Two-tailed *p* value
1	Treated	SBP	Pre	19	141.52 (14.99)	23.47	<0.001^*∗∗*^
			Post	19	118.05 (9.46)		
2	Untreated	SBP	Pre	13	146.69 (19.12)	27.03	<0.001^*∗∗*^
			Post	12	119.66 (9.09)		
3	Treated	DBP	Pre	19	86.94 (11.11)	11.52	0.001^*∗∗*^
			Post	19	75.42 (6.86)		
4	Untreated	DBP	Pre	13	95.15 (7.67)	15.57	0.004^*∗∗*^
			Post	12	79.58 (5.05)		

SD, standard deviation. ^*∗∗*^Highly significant (*p* value < 0.005).

**Table 4:** Comparison of responders to yoganidra (SBP ≥10 mm hg and DBP≥ 5 mm hg) among treated and untreated cases.

Sl. no.	Parameters	Treated group	Untreated group
1	SBP responders (≥10 mm hg)	18/19 (94.73%)	10/12 (83.33)
2	SBP nonresponders (<10 mm hg)	1/19 (5.26%)	2/12 (16.66)
3	DBP responders (≥5 mm hg)	15/19 (78.94%)	12/12 (100)
4	DBP nonresponders (<5 mm hg)	4/19 (21.05%)	0

## Data Availability

The datasets for the current study are available from the corresponding author upon request.

## Supplementary Materials

Supplementary Materials Weekly blood pressure was recorded for experimental group subjects, and the mean and SD of both SBP and DBP are as shown in Supplementary Table S1. A gradual reduction in both SBP and DBP was observed among the intervention group.
