# Supplementary material for: Effect of Yoganidra on Blood Pressure, Hs-CRP, and Lipid Profile of Hypertensive Subjects: A Pilot Study
Source: Evid Based Complement Alternat Med. 2021 Dec 30;2021:2858235. doi: 10.1155/2021/2858235 (PMC8739171; doi:10.1155/2021/2858235)
Supplement: Supplementary Materials — Weekly blood pressure was recorded for experimental group subjects, and the mean and SD of both SBP and DBP are as shown in Supplementary Table S1. A gradual reduction in both SBP and DBP was observed among the intervention group. [file 2858235.f1.docx]

**Supplementary Table (S1):** Weekly Mean and SD of SBP and DBP

| **Weeks** | **SBP** | | **DBP** | |
| --- | --- | --- | --- | --- |
|  | Mean(mm Hg) | ±SD | Mean(mm Hg) | ±SD |
| 1 | 142.9 | 16.46 | 90.28 | 10.42 |
| 2 | 135.8 | 12.86 | 85.90 | 8.62 |
| 3 | 132.83 | 14.74 | 83.74 | 8.56 |
| 4 | 130.32 | 13.67 | 82.38 | 7.17 |
| 5 | 131.90 | 12.32 | 83.70 | 8.55 |
| 6 | 128.61 | 16.20 | 82.90 | 8.26 |
| 7 | 126.96 | 16.11 | 80.48 | 8.36 |
| 8 | 125.51 | 15.58 | 80.96 | 8.47 |
| 9 | 125.35 | 15.63 | 79.70 | 8.60 |
| 10 | 126.45 | 15.75 | 81.38 | 8.42 |
| 11 | 125.12 | 15.49 | 80.61 | 8.32 |
| 12 | 118.68 | 9.21 | 77.03 | 10.42 |

**SBP:** Systolic Blood Pressure **DBP: D**iastolic Blood Pressure
